# Cytarabine Pharmacogenomics and Outcomes Among Children and Young Adults With Acute Myeloid Leukemia

**DOI:** 10.1001/jamanetworkopen.2025.16296

**Published:** 2025-06-23

**Authors:** Richard J. Marrero, Vivek M. Shastri, Deedra Nicolet, Krzysztof Mrózek, Christopher J. Walker, William G. Blum, Bayard L. Powell, Jonathan E. Kolitz, Joseph O. Moore, Geoffrey L. Uy, Wendy Stock, Andrew J. Carroll, John C. Byrd, Richard Aplenc, Todd M. Cooper, Alan S. Gamis, Huiyun Wu, Stanley Pounds, Yi-Cheng Wang, Todd A. Alonzo, Soheil Meshinchi, Ann-Kathrin Eisfeld, Edward A. Kolb, Jatinder K. Lamba

**Affiliations:** 1 Department of Pharmacotherapy and Translational Research, University of Florida College of Pharmacy, Gainesville; 2 The Clara D. Bloomfield Center for Leukemia Outcomes Research, The Ohio State University Comprehensive Cancer Center, Columbus; 3 Winship Cancer Institute of Emory University, Atlanta, Georgia; 4 Wake Forest Baptist Comprehensive Cancer Center, Winston-Salem, North Carolina; 5 Zucker School of Medicine at Hofstra/Northwell, Zuckerberg Cancer Center, Lake Success, New York; 6 Duke University Medical Center, Durham, North Carolina; 7 Washington University School of Medicine, Saint Louis, Missouri; 8 University of Chicago Medicine, Chicago, Illinois; 9 Department of Medicine, Section of Hematology/Oncology, University of Chicago, Chicago, Illinois; 10 Department of Genetics, University of Alabama at Birmingham; 11 Department of Internal Medicine, University of Cincinnati, Cincinnati, Ohio; 12 Division of Oncology, Children’s Hospital of Philadelphia, Philadelphia, Pennsylvania; 13 Division of Pediatric Hematology, Oncology, Bone Marrow Transplant & Cellular Therapy, Seattle Children’s Hospital, Seattle, Washington; 14 Hematology/Oncology/Bone Marrow Transplant, Children’s Mercy Kansas City, Kansas City, Missouri; 15 Department of Biostatistics, St Jude Children’s Research Hospital, Memphis, Tennessee; 16 Children’s Oncology Group, Monrovia, California; 17 University of Southern California Keck School of Medicine, Los Angeles; 18 Fred Hutchinson Cancer Center, Seattle, Washington; 19 Leukemia Lymphoma Society, Bronx, New York; 20 University of Florida Health Cancer Center, University of Florida, Gainesville

## Abstract

**Importance:**

Therapeutic responses in acute myeloid leukemia (AML) demonstrate considerable variability both across and within established risk stratifications and age groups. Moreover, significant racial disparities persist, with Black patients experiencing inferior survival outcomes compared with their White counterparts.

**Objective:**

To validate the association of the previously reported 10 single nucleotide variant (SNV)–based ara-C pharmacogenomics score (ACS10) with survival outcomes in a large cohort of pediatric AML patients; to evaluate whether ACS10 remains relevant in an adolescent and young adult (AYA) population of patients with AML treated with similar intensive induction chemotherapy protocols; and to assess the association of ACS10 with race and treatment outcomes in both cohorts.

**Design, Setting, and Participants:**

This cohort study included patients from the Children’s Oncology Group’s AAML1031 trial, a multicenter, open-label randomized clinical trial that enrolled pediatric patients with newly diagnosed, treatment-naive primary AML from June 2011 to July 2017 (aged 0 to 29.5 years) and from the Alliance for Clinical Trials in Oncology frontline protocols, which included AYA patients from 9 different trials that enrolled patients with newly diagnosed AML from 1992 to 2010. Data were analyzed from September 2022 to March 2025.

**Exposures:**

Patients in the AAML1031 trial were randomized to 2 arms, standard chemotherapy alone or standard chemotherapy with the addition of bortezomib. Patients in the Alliance for Clinical Trials in Oncology cohorts were treated with similar intensive induction chemotherapy protocols.

**Main Outcomes and Measures:**

ACS10 scores were evaluated for association with outcomes according to race, treatment arm, and hematopoietic stem cell transplant (HSCT) status.

**Results:**

The study included 1086 patients with AML. There were 717 patients from the pediatric AML cohort (median [range] age, 9.6 [0.04-29.2 years]; 379 [53%] male; 33 [5%] Asian, 84 [12%] Black, and 522 [73%] White) and 369 AYA patients with AML from the Alliance for Clinical Trials in Oncology group (median [range] age, 30 [17-39] years; 196 [53%] male; 7 [2%] Asian, 32 [9%] Black, and 288 [78%] White). Within the standard treatment arm of AAML1031, patients in the low ACS10 group had significantly worse event-free survival (EFS) compared with those in the high ACS10 group (all patients: hazard ratio [HR], 1.42; 95% CI, 1.05-1.95; *P* = .02; non-HSCT cohort: HR, 1.48; 95% CI, 1.06-2.07; *P* = .02). The ACS10 score remained significantly associated with EFS in multivariable analysis after adjusting for age, race, risk group and white blood cell count, within the standard treatment arm (HR, 1.44; 95% CI, 1.03-2.02; *P* = .03). In the Alliance for Clinical Trials in Oncology AYA non-HSCT cohort, the low ACS10 score group had significantly inferior overall survival (OS) and a higher point estimate for EFS compared with patients with a high ACS10 score (OS: HR, 1.50; 95% CI, 1.05-2.14; *P* = .03; EFS: HR, 1.32; 95% CI, 0.95-1.83; *P* = .10). A higher number of early deaths was observed in the low ACS10 group compared with the high ACS10 group, but the difference was not statistically significant (death within 30 days of treatment initiation: 6 of 112 [5%] vs 2 of 257 [1%]; *P* = .07). Across both cohorts, a low ACS10 score was significantly more abundant in Black patients compared with White patients (eg, in Alliance for Clinical Trials in Oncology cohort, 27 of 32 Black patients [84%] had low ACS10 scores compared with 64 of 288 White patients [22%]; *P* < .001) and inferior survival was observed in Black patients (eg, OS of Black compared with White patients in AAML1031 cohort: HR, 1.47; 95% CI, 1.02-2.13; *P* = .04). In the AAML1031 cohort, there were no significant differences in EFS or OS between Black and White patients receiving augmented treatment, suggesting that the addition of bortezomib was associated with benefit for Black patients.

**Conclusions and Relevance:**

In this study of 717 pediatric and 369 AYA patients with AML, the ACS10 score was associated with EFS in pediatric and AYA patients when treated with a standard induction regimen. There was a higher abundance of low ACS10 scores in Black patients, and Black patients treated with augmented therapy (ie, the addition of bortezomib) seemed to have improved outcomes. Integrating the ACS10 score into a prospective clinical trial to personalize induction therapy based on an individual’s genetic profile has the potential to improve treatment outcomes.

## Key Points

**Question:**

Is the ara-C pharmacogenomics score (ACS10), a pharmacogenomics score for pediatric patients with acute myeloid leukemia, clinically relevant in adolescent and young adult patients?

**Findings:**

In this cohort study with 1086 patients, a low ACS10 score was associated with inferior event-free survival in pediatric, adolescent, and young adult patients when treated with a standard induction regimen. There was a higher abundance of low ACS10 scores in Black patients, but the results suggested that observed racial difference could be overcome by therapy augmentation, such as the addition of bortezomib.

**Meaning:**

These findings hold promise for personalizing an induction regimen, guided by ACS10 score, and further developing strategies to mitigate observed outcome differences in acute myeloid leukemia by race.

## Teaser

This cohort study evaluates whether the ara-C pharmacogenomics score is associated with survival outcomes among pediatric, adolescent, and young adult patients with acute myeloid leukemia (AML) and whether the score could help personalize treatment for this population.

## Introduction

The overall prognosis for acute myeloid leukemia (AML) is poor, with disease heterogeneity, relapse and refractory disease, and treatment-related toxic effects posing significant challenges in treating AML.^1^ Although initial induction chemotherapy regimens often lead to remission in the majority of pediatric cases, a significant number of these patients ultimately succumb to the disease due to relapse or refractory AML.^2^ For more than 5 decades, frontline treatment of AML has consisted of cytarabine (ara-C) in combination with anthracyclines.^3^ Ara-C is a prodrug that requires activation to ara-C triphosphate (ara-CTP), with treatment efficacy associated with the intracellular levels of the active drug.^4^ To identify genetic factors associated with ara-C’s response, we recently reported a pharmacogenomics score (ACS10) consisting of 10 single nucleotide variants (SNVs) in 9 ara-C metabolic pathway genes (eTable 1 in Supplement 1).^5^ In pediatric patients treated with standard chemotherapy in the multisite AML02 and Children’s Oncology Group (COG) AAML0531 trials, a low ACS10 score (≤0) was associated with poor event-free survival (EFS) and overall survival (OS) compared with patients with a high ACS10 score (>0). Low ACS10 score was more abundant in Black patients than in White patients.^5^ This observed racial disparity in AML outcomes is interesting, especially when considering that prior reports have shown Black patients have inferior survival compared with non-Hispanic White patients across pediatric and adolescent and young adult (AYA) populations.^6,7,8,9,10,11,12,13,14,15^ In pediatric patients with AML, a report from COG^15^ showed inferior OS in Hispanic and Black patients compared with White patients. According to another report, which used the Therapeutically Applicable Research to Generate Effective Treatments (TARGET) database, there was a greater prevalence of t(8;21)(q22;q22) in Black and Hispanic patients and a higher occurrence of rearrangements involving 11q23 and the lysine methyltransferase 2a gene (*KMT2A*) in Black patients compared with non-Hispanic White patients.^8,10^ Notably, inferior survival was observed in Black patients with 11q23/*KMT2A* rearrangements and in those with core-binding factor AML (ie, with t[8;21] and inv[16][p13.1q22]).^10^ For AYA patients with AML (aged 18 to 39 years) treated in Alliance for Clinical Trials in Oncology (hereafter, Alliance) trials, higher early death rates, lower complete remission (CR) rates, and nearly a decade shorter median OS was reported for Black patients.^8^ Given the association of ACS10 score groups with outcomes and their differential abundance across races, the objectives of this analysis were (1) to validate the association of ACS10 with outcomes in a large and independent cohort of 717 patients treated in the COG AAML1031 trial; (2) to evaluate whether ACS10 is associated with outcomes in an AYA population of patients with AML treated with similar intensive induction chemotherapy on Alliance frontline protocols; and (3) to evaluate the contribution of the pharmacogenomics-based ACS10 score to observed racial disparities in outcomes via dedicated survival analyses Black and White patients.

## Methods

The study was approved by the institutional review board each participating institution, and informed consent was obtained from parents, guardians, or patients and assents from the patients, as appropriate, in accordance with the approved clinical trial protocols. This report adheres to the Strengthening the Reporting of Genetic Association Studies (STREGA) reporting guidelines for the proposed work validating the ACS10 score in AML.^28^

### Patient Cohorts

#### Cohort 1: Pediatric Cohort From AAML1031 Trial

The current analysis included 717 pediatric and AYA patients with AML (age range, 0.04-29 years) with both molecular genetics and clinical outcome data available who were treated in the multicenter AAML1031 trial (NCT01371981). Details of study design, treatment arms, eligibility, and clinical outcome have been previously reported.^16^ Overall, the AAML1031 trial enrolled newly diagnosed patients who received either conventional AML treatment (ara-C, daunorubicin, and etoposide [ADE]; arm A) or conventional treatment with the addition of bortezomib (ADE + bortezomib; arm B). Of note, patients with an internal tandem duplication of the *FLT3* gene were treated on arm C and were not included in this analysis. Risk classification was based on cytogenetics, molecular markers, and measurable residual disease (MRD) after the initial induction regimen. Of note, protocol-based classification included MRD after induction 1 and divided patients into low- or high-risk groups; however, for this analysis, we utilized diagnostic cytogenetic and molecular features to classify patients into risk groups (high, standard, and low) using 2022 European LeukemiaNet (ELN) classification.^17^ Four courses of chemotherapy were given to low-risk patients or high-risk patients without an appropriate donor. High-risk patients with an appropriate donor received 3 courses of chemotherapy followed by allogeneic hematopoietic stem-cell transplant (HSCT). The AAML1031 trial showed no benefit for adding bortezomib to the standard chemotherapy induction regimen.

#### Cohort 2: AYA Cohort From the Cancer and Leukemia Group B/Alliance

A total of 369 newly diagnosed AYA patients with AML (age 17-39 years) similarly treated on 9 Alliance frontline protocols were included in the analysis.^18,19,20,21,22,23,24,25,26^ Briefly, patients with acute promyelocytic leukemia, AML secondary to myelodysplastic syndromes, and patients with therapy-related AML were excluded. Patients removed from protocol treatment to undergo allogeneic hematopoietic cell transplantation in complete remission after induction 1 were excluded from the analysis. All patients had centrally reviewed cytogenetics, targeted gene alteration profiling of 80 leukemia-associated genes as well as micro-array-based SNV data available.^27^ Of note, the Cancer and Leukemia Group B (CALGB) is now part of the Alliance.

### Clinical Outcome End Points

EFS was defined as the time from study entry until death, refractory disease, or relapse of any type, whichever occurred first. OS was defined as time from study entry until death, with living patients censored on the date of last follow-up.

### Reporting Race and Ethnicity

For the AAML1031 clinical trial cohort, demographic information on race and ethnicity was provided by patients or their legal guardians at the time of study enrollment. Race and ethnicity categories were African American or Black, American Indian or Alaska Native, Asian, multiple races, Native Hawaiian or Other Pacific Islander, White, or unknown. Ethnicity, collected in the same manner, consisted of the following groups: Hispanic or Latino, not Hispanic or Latino, or unknown. Similarly, for the CALGB/Alliance protocol cohorts, race was classified as Asian, Black, or White. In this study, racial categories not classified as Asian, Black, or White were consolidated under the “other” designation. This was done to retain a large enough sample of patients who did not belong to the Asian, Black, or White groups to run meaningful multivariable analyses. This information was obtained to define the baseline demographic profiles of participants enrolled in the clinical trials and to assess potential associations with clinical outcomes in this study.

### Genotyping

For the AAML1031 cohort, genomic DNA from 717 patients was genotyped for 10 previously defined ACS10 SNVs using TaqMan Allelic Discrimination assays, as per previous report.^5^ For the Alliance cohorts, ACS10 SNVs were extracted from previously published genotyping data that was performed by deCODE Genetics using the Infinium Omni-1 Quad-bead array (Illumina), with additional genotype imputation as per previous report.^27^ The ACS10 score was calculated for each patient using the previously defined equation (high ASC10, >0; low ACS10, ≤0) for both cohorts and is described in eTable 1 in Supplement 1.

### Statistical Analysis

The Wilcoxon rank-sum test and Kruskal-Wallis test were used to compare medians of numeric variables across groups and χ^2^ and Fisher exact tests were used to evaluate the association among pairs of categorical variables. EFS and OS probabilities for ACS10 score groups were estimated using the Kaplan-Meier method. Cox proportional hazard regression models were used to evaluate the associations of ACS10 score with EFS and OS. The 95% CI of hazard ratios (HRs) was calculated to quantitatively measure the association with clinical outcome. Analyses accounting for transplant used Cox regression models with transplant as a time-varying covariate for estimation and testing of HRs and used the method of Jay and Betensky^29^ to visualize survival outcomes in Kaplan-Meier style plots. Significance levels for the association of ACS10 score with clinical outcome were set at *P* < .05. All statistical analyses were performed using R version 4.3.1 (R Project for Statistical Computing).

## Results

### AAML1031 Cohort

Among the 717 patients from AAML1031 trial, the median (range) age was 9.6 (0.04-29.2) years; 370 patients (53%) were male; 35 (5%) were Asian, 84 (12%) were Black, and 522 (73%) were White; and 334 (47%) were treated with standard ADE (arm A), and 383 (53%) were treated with ADE + bortezomib (arm B). eTable 2 in Supplement 1 summarizes the characteristics of the patients in this cohort. Overall, among the 717 patients from AAML1031 study, 369 events were reported, including 276 relapses and 230 deaths that occurred within 5 years. Prior to testing ACS10 score, we evaluated survival by treatment arms (ADE vs ADE + bortezomib), and consistent with the results of the trial,^16^ no significant difference in EFS or OS was observed in the 717 patients with AML included in this analysis (eFigure 1 in Supplement 1). The ACS10 score was computed for 717 patients as described previously^5^ and is summarized in eTable 2 in Supplement 1. Patients were classified into 2 groups, with 249 patients (35%) in the low ACS10 score (≤0) group and 468 patients (65%) in the high ACS10 score (>0) group (eFigure 2 in Supplement 1). Age, sex, cytogenetics, and white blood cell (WBC) count did not differ significantly by score groups; however, ACS10 score groups differed by race and HSCT status (eTable 2 in Supplement 1). Thus, the analysis was done in the whole cohort and in patients who did not receive HSCT.

#### Outcome by ACS10 Score at the Whole Cohort Level

At the cohort level (arms A and B combined) in univariate analysis, ACS10 score was not significantly associated with OS and EFS; however, for EFS, a low ACS10 score had a higher point estimate compared with a high ACS10 score (HR, 1.19; 95% CI, 0.98-1.48; *P* = .09) (eFigure 3A in Supplement 1). Similar results were obtained when restricting the analysis to the non-HSCT group; patients with a low ACS10 score had a higher point estimate in EFS compared with those with a high ACS10 score (HR, 1.22; 95% CI, 0.97-1.53; *P* = .08) (eFigure 3C in Supplement 1). No difference in OS was observed (eFigure 3B and 3D in Supplement 1). In the whole cohort, 5-year EFS estimates were 44% for the low ACS10 score group and 50% for the high ACS10 score group. Further analysis, adjusting for transplant as a time-dependent covariate, found that patients with a low ACS10 score had inferior EFS compared with those with a high ACS10 score (all patients: HR, 1.25; 95% CI, 1.02-1.56; *P* = .04; standard and high molecular risk patients: HR, 1.30; 95% CI, 1.01-1.67; *P* = .04).

#### Outcome by ACS10 Score Within Treatment Arms A and B

**Figure 1.**
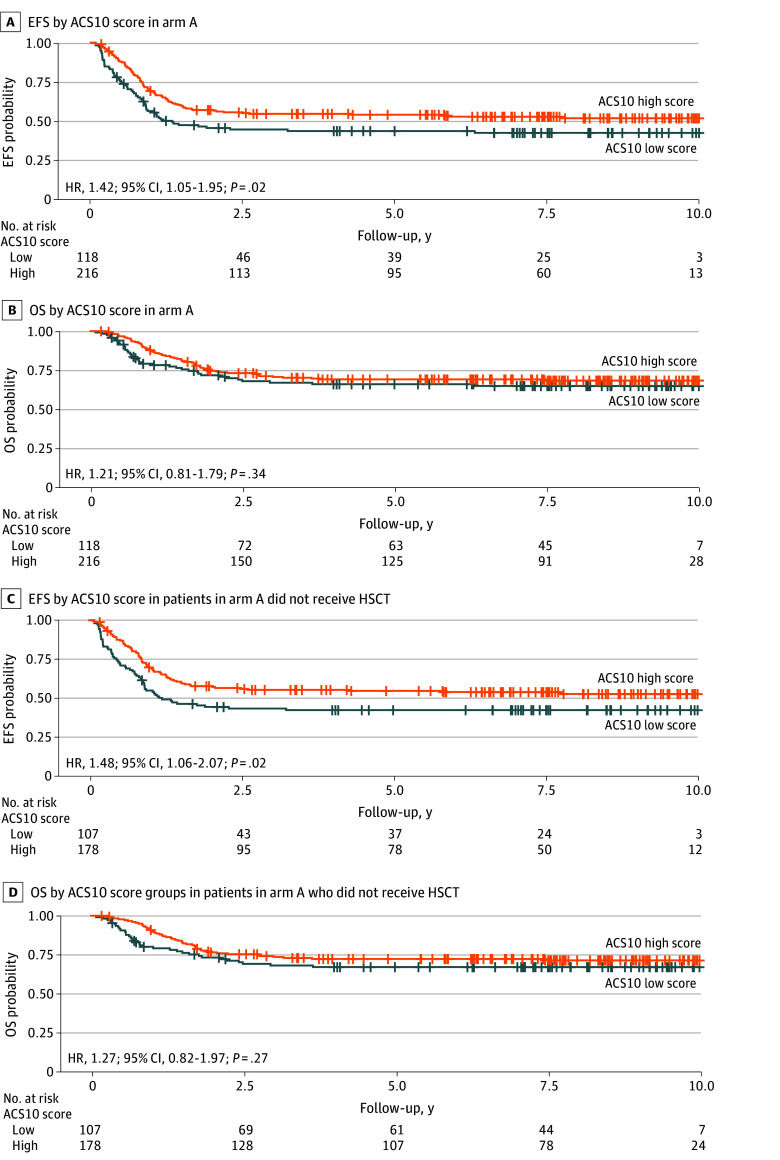
Kaplan-Meier Survival Curves by Ara-C Pharmacogenomics Score (ACS10) Group in the AAML1031 Trial Event-free survival (EFS) by ACS10 score in the standard chemotherapy arm A (A) and in arm A among patients who did not receive hematopoietic stem cell transplant (HSCT) (C); overall survival (OS) by ACS10 score in the standard chemotherapy arm A (B) and in arm A among non-HSCT patients (D). A high ACS10 score was used as the reference group.

Analysis within each treatment arm by ACS10 score group showed that within the standard ADE treatment arm (arm A), patients with a low ACS10 score had significantly worse EFS than those with a high ACS10 score (HR, 1.42; 95% CI, 1.05-1.95; *P* = .02) (Figure 1A), but no significant difference in OS was observed (HR, 1.21; 95% CI, 0.81-1.79; *P* = .34) (Figure 1B). Five-year EFS estimates in arm A were 41% for the low ACS10 score group and 52% for the high ACS10 score group. Restricting the sample to the non-HSCT group showed consistent results, with inferior EFS in the low ACS10 score group compared with the high ACS10 score group (HR, 1.48; 95% CI, 1.06-2.07; *P* = .02) (Figure 1C). Five-year EFS estimates for patients who did not receive HSCT treated on arm A were 40% for the low ACS10 score group and 53% for the high ACS10 score group. OS was not significantly different by ACS10 group within arm A (HR, 1.27; 95% CI, 0.82-1.97; *P* = .27) (Figure 1D). Within arm B (ADE + bortezomib), we did not observe any difference in EFS or OS by ACS10 score groups in all patients or by restricting the sample to the non-HSCT group (eFigure 4 in Supplement 1). Further analyses adjusting for transplant as a time-dependent covariate in the combined treatment cohort (arm A and arm B) showed significantly worse EFS for the low ACS10 score group compared with the high ACS10 score group, both in the overall population and among those with standard or high-risk molecular features (eFigure 5A and B in Supplement 1). Within arm A, worse EFS was observed for the low ACS10 score group than for the high ACS10 score group at the cohort level but not when stratified by molecular risk groups (all patients: HR, 1.49; 95% CI, 1.09-1.78; *P* = .01; patients with standard and high molecular risk: HR, 1.41; 95% CI, 0.97-2.06; *P* = .07) (eFigure 5C and D in Supplement 1). Furthermore, we observed no difference in EFS outcome by ACS10 score using HSCT as a time varying covariate for all patients in arm B (HR, 1.09; 95% CI, 0.81-1.45; *P* = .59) (eFigure 5E in Supplement 1); however, for the standard and high molecular risk group in arm B, significantly reduced EFS was observed for the low ACS10 score group compared with the high ACS10 score group (HR, 1.26; 95% CI, 1.02-1.56; *P* = .04) (eFigure 5F in Supplement 1).

**Figure 2.**
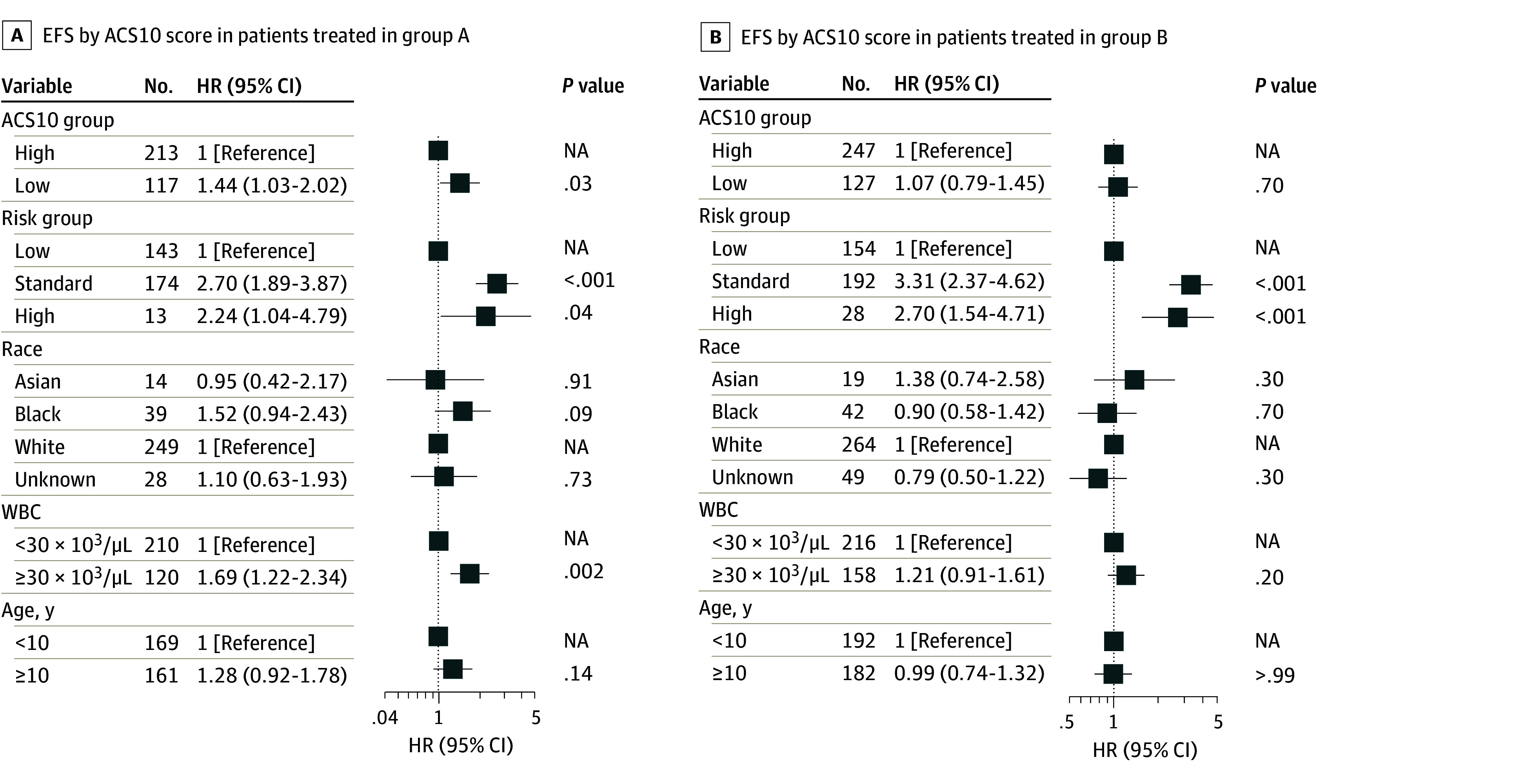
Event-Free Survival (EFS) by Ara-C Pharamacogenomics Score (ASC10) HR indicates hazard ratio; NA, not applicable; WBC, white blood cells.

In multivariable analysis, adjusting for known prognostic factors, such as risk group, race, age, and WBC count, similar associations were observed, with a low ACS10 score remaining significantly associated with EFS in arm A (Figure 2A) but not in the augmented arm B (Figure 2B). No significant difference in OS was observed (eFigure 6 in Supplement 1).

These results indicated a potential for a statistical interaction between arm and ACS10 score on outcomes. We formally evaluated for such an interaction by fitting a Cox model of EFS with arm, the numeric ACS10 score, and the arm × ACS10 score interaction on the data for the entire AAML1031 cohort. In this analysis, the rate of EFS events for arm B was 0.97 times that for arm A (HR, 0.97; 95% CI, 0.75-1.24; *P* = .80); each unit increase in the ACS10 score modified the EFS event rate by a factor of 0.92 in arm A (HR, 0.92; 95% CI, 0.83-1.01; P = .08) and a factor of 1.01 in arm B (HR, 1.01; 95% CI, 0.92-1.10; *P *for interaction = .15). Thus, consistent with the previously described results, this analysis indicates that EFS is nominally, but not statistically significantly, improved with increasing ACS10 score in arm A but that ACS10 and EFS are not associated in arm B.

Overall toxic effects observed in the AAML1031 clinical trial have been described in detail elsewhere.^16^ Briefly, most toxic effects, including infectious complications, kidney toxic effects, and declines in ejection fraction, did not significantly differ between treatment arms. However, during the first induction, patients in the ADE + bortezomib arm had a higher incidence of peripheral neuropathy and respiratory distress syndrome. We assessed the association between ACS10 score and toxic effects during induction 1. Among the patients who experienced grade 3 or greater toxic effects within induction 1, 342 patients (66%) of the patients had high ACS10 score and 176 (34%) had low ACS10 score.

#### Outcomes by Race

**Figure 3.**
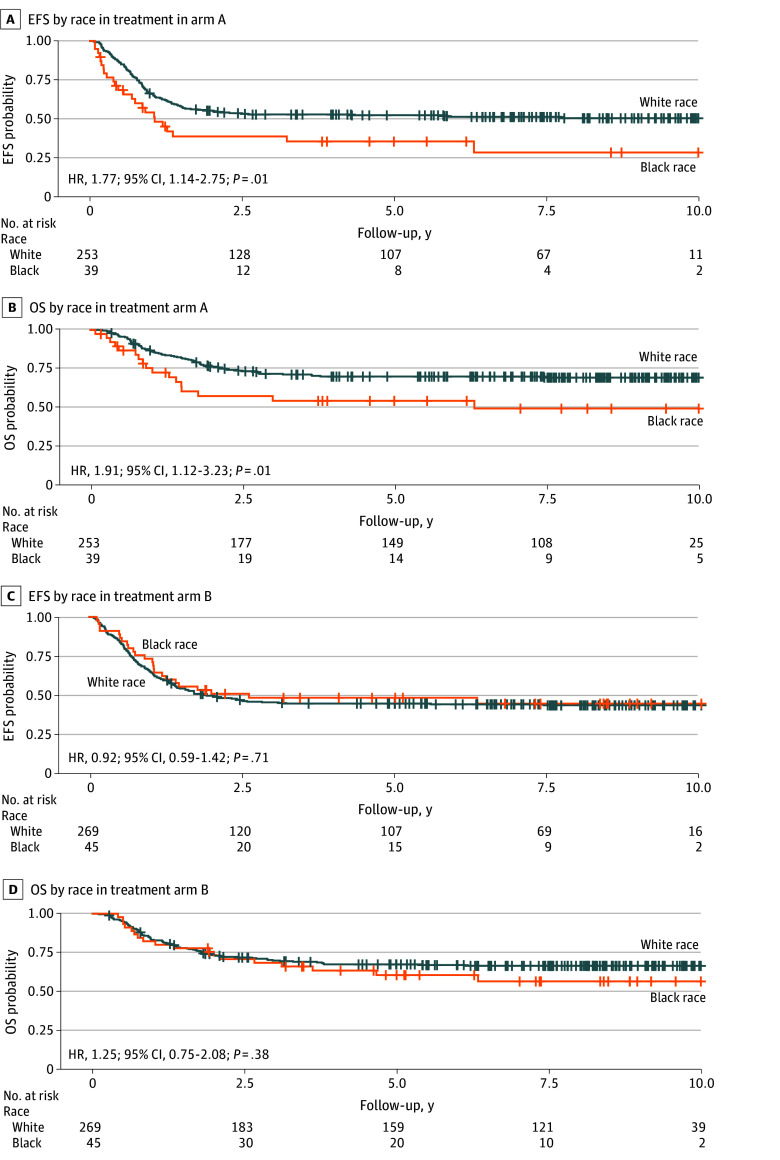
Kaplan-Meier Survival Curves Within Each Treatment Arm by Race Event-free survival (EFS) by race in treatment arm A (A) and arm B (C); overall survival (OS) by race in treatment arm A (B) and arm B (D). White patients were used as the reference group.

At the whole cohort level (arm A and arm B), the 84 Black patients had worse OS than the 522 White patients (HR, 1.47; 95% CI, 1.02-2.13; *P* = .04); the difference in EFS was not statistically significant (HR, 1.24; 95% CI, 0.89-1.67; *P* = .21). Within arm A, 39 Black patients had reduced EFS and OS compared with 253 White patients (EFS: HR, 1.77; 95% CI, 1.14-2.75; *P* = .01; OS: HR, 1.91; 95% CI, 1.12-3.23; *P* = .02) (Figure 3A and B). In contrast, no significant differences in EFS and OS were observed between Black and White patients in arm B, implying that the addition of bortezomib provided benefit to Black patients (Figure 3C and D). Further comparisons of EFS among Black patients in arm A vs arm B showed a lower point estimate in EFS for those within arm B, but the results were not statistically significant (HR, 0.62; 95% CI, 0.35-1.07; *P* = .10) (eFigure 7 in Supplement 1).

#### Outcomes by Race and ACS10 Scores

**Figure 4.**
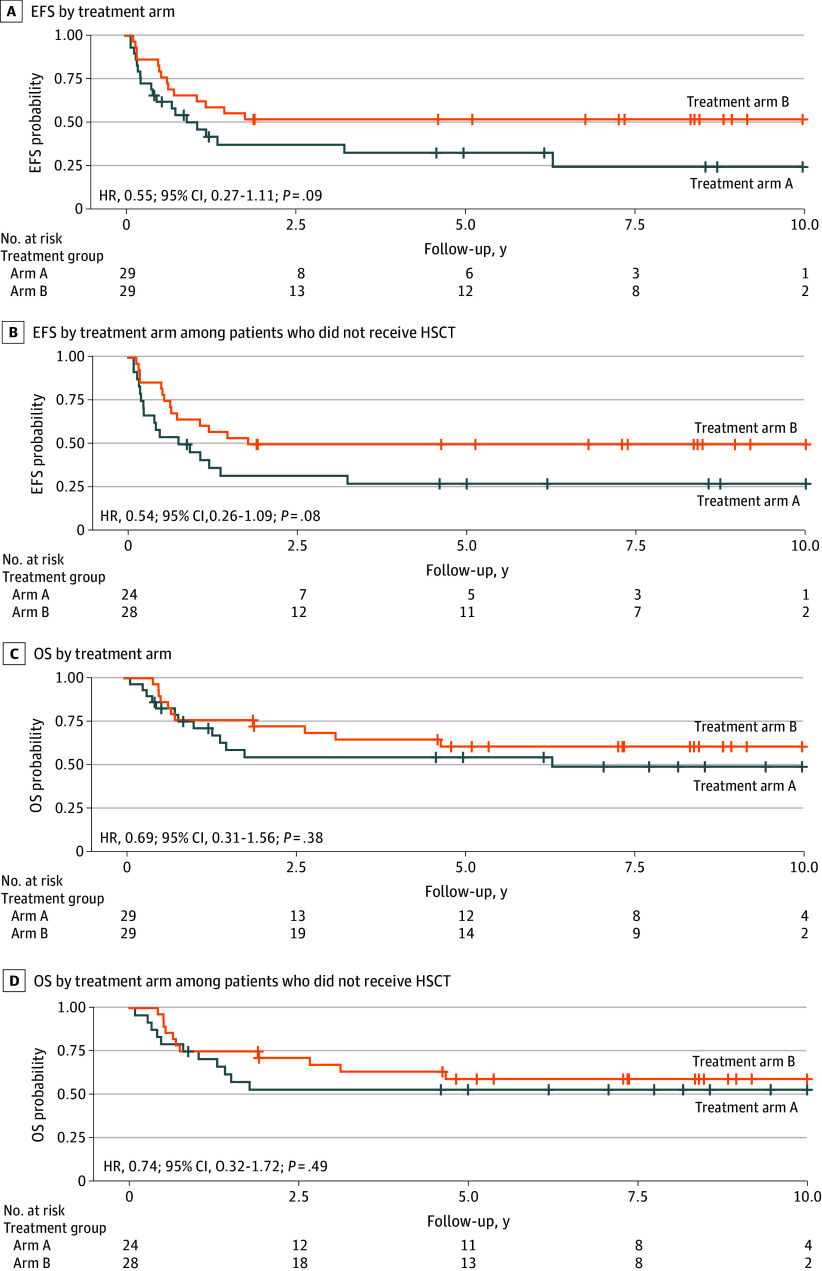
Kaplan-Meier Survival Curves by Treatment Arm in Black Patients With Acute Myeloid Leukemia and a Low Ara-C Pharmacogenomics Score (ACS10) Event-free survival (EFS) at the cohort level (A) and among patients who did not receive hematopoietic stem cell transplant (HSCT) (B). Overall survival (OS) at the cohort level (C) and among patients who did not receive HSCT. Treatment arm A was used as the reference group.

Consistent with our previous investigation, more Black patients had low ACS10 scores than White patients (Black: 58 of 84 [69%]; White: 144 of 522 [28%]; *P* < .001) (eTable 2 and eFigure 2B and C in Supplement 1). Due to the small number of Black patients with a high ACS10 score in arm A, we restricted our analysis to the low ACS10 score group. Black patients did not experience a significant improvement in EFS when treated with ADE plus bortezomib (arm B) compared with standard arm A at both the cohort level and among those who did not receive HSCT (all patients: HR, 0.55; 95% CI, 0.27-1.11; *P* = .09; non-HSCT: HR, 0.53; 95% CI, 0.26-1.09; *P* = .08) (Figure 4A and B). There was no difference for OS among Black patients with a low ACS10 score (all patients: HR, 0.69; 95% CI, 0.31-1.56; *P* = .38; non-HSCT: HR, 0.74; 95% CI, 0.32-1.72; *P* = .49) (Figure 4C and D).

### AYA Patients (CALGB/Alliance)

For 369 patients from Alliance trials, 196 (53%) were male; 7 (2%) were Asian, 32 (9%) were Black, and 288 (78%) were White; and the median (range) age was 30 (17-39) years. eTable 3 in Supplement 1 summarizes the characteristics of the patients within this cohorts. Overall, among 369 patients, 87 CRs, 148 relapses, and 205 deaths were reported. The ACS10 score was calculated for each patient, and 257 patients (70%) were classified with high ACS10 scores and 112 patients (30%) with low ACS10 scores. There was no difference in age or 2022 ELN risk group distribution among patients within ACS10 score groups; however, ACS10 score group distribution differed by race, with 27 of 32 Black patients (84%) belonging to the low ACS10 score group compared with only 64 of 288 White patients (22%). While the number of Hispanic patients was small (21 patients), their ACS10 score distribution resembled that of White patients, with 13 Hispanic AYA patients (38%) belonging to the low ACS10 score group. Patient characteristics in whole cohort and in score groups are summarized in eTable 3 in Supplement 1.

#### Cohort-Level Outcomes by ACS10 Score

**Figure 5.**
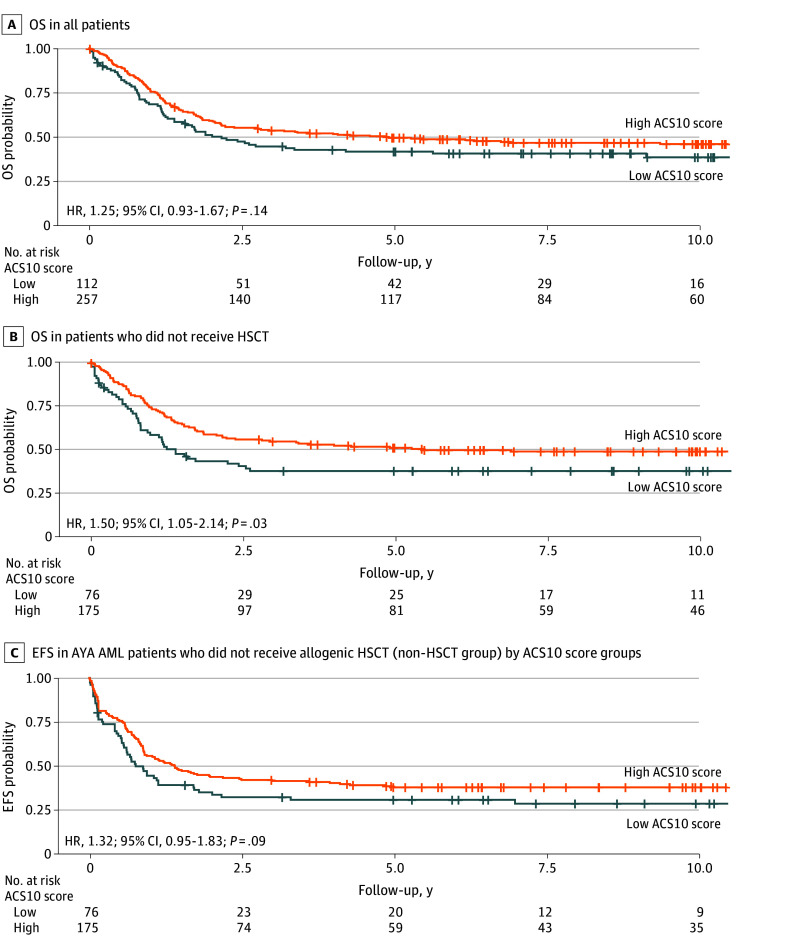
Kaplan-Meier Survival Curves for Overall Survival (OS) and Event-Free Survival (EFS) in Patients with Acute Myeloid Leukemia Enrolled in Alliance for Clinical Trials in Oncology Trials by Ara-C Pharmacogenomics Score (ACS10) A, OS in all patients by high and low ACS10 score groups. B, OS in patients who did not receive allogeneic hematopoietic stem cell transplant by ACS10 score groups. C, EFS in patients who did not receive allogenic HSCT. A high ACS10 score was used as the reference group.

Within the whole cohort, patients with a low ACS score had a higher point estimate for OS compared with patients with a high ACS10 score (HR, 1.25; 95% CI, 0.93-1.67; *P* = .14) (Figure 5A). Among patients who never received an allogeneic HSCT during their treatment, patients with a low ACS10 score had shorter OS compared with patients with a high ACS10 score (HR, 1.50; 95% CI, 1.05-2.14; *P* = .03; 3-year OS rates: 38% [95% CI, 27%-49%] vs 55% [95% CI, 47% 62%]) (Figure 5B). The median OS was 1.4 years for the low ACS10 score group compared with 5.5 years for the high ACS10 score group. Furthermore, patients with a low ACS10 score a higher point estimate for EFS than those with a high ACS10 score (HR, 1.32; 95% CI, 0.95-1.83; *P* = .10; 3-year EFS rates, 32% [95% CI, 22%-43%] vs 42% [95% CI, 34%-49%]) (Figure 5C); additionally, those with a low ACS score had a higher number of early deaths than those with a high ACS score (death within 30 days of treatment initiation; 6 of 122 [5%] vs 3 of 257 [1%]; *P* = .07), although the difference was not statistically significant.

#### Outcome by ACS10 Score and Race

Significant differences in ACS10 score groups by race were found in the Alliance cohort, with 27 of 32 Black patients (84%) belonging to the low ACS10 score group compared with 64 of 288 White patients (22%) (*P* < .001). Furthermore, survival of Black patients within this cohort was lower than that of White patients (whole cohort: (HR, 1.29; 95% CI, 1.03-1.60; *P* = .02; low ACS10 score group: HR, 1.38; 95% CI, 1.05-1.81; *P* = .02). However, despite this being one of the larger studies with data from 9 clinical trials, the sample size of Black patients was relatively small (27 with low ACS10 scores and 5 with high ACS10 scores) and precluded a sufficiently powered analysis.

## Discussion

For over 5 decades cytarabine has been the backbone of AML treatment and will likely remain so for the foreseeable future. Currently, it is administered to patients at varying dosages and regimens without accounting for any genetic or racial parameters. Racial disparities in the survival of patients with AML are also well established, with reports showing Black pediatric, AYA, and adult patients have inferior outcomes compared with White patients.^6,7,8,9,10,11,12,13,14,15^ Previous work by Plunkett and colleagues^4^ demonstrated a strong association of intracellular ara-CTP levels with achievement of CR. In our prior publication,^30^ we reported on SNVs in cytarabine pathway genes associated with intracellular ara-CTP levels, and 4 of the SNVs in the current ACS10 score were part of that study. One of the major challenges in conducting such analyses in retrospective studies is the lack of early posttreatment bone marrow samples, which are typically not collected soon after initiation of induction treatment. In prospective studies, obtaining bone marrow samples between 24 to72 hours after initiation of cytarabine is also not part of standard clinical practice. Future studies focusing on the utility of peripheral blood samples obtained 24 to 48 hours after ara-C infusion may provide a more feasible alternative for assessing intracellular ara-CTP levels. In the interim, we analyzed the association between ACS10 score and intracellular ara-CTP levels in patients from the AML97 clinical trial,^31^ where cellular ara-CTP levels were quantitated in specimens obtained 24 hours after initiation of ara-C infusion. Our analysis found that patients with low ACS10 scores had lower intracellular ara-CTP levels^30^ (eFigure 8 in Supplement 1). A low ACS10 score (≤0) was associated with reduced activation and with poor EFS and OS in patients treated with standard chemotherapy in AML02 and AAML0531 trials. However, augmentation of standard induction therapy with high-dose ara-C (in the AML02 trial) or addition of gemtuzumab ozogamicin (in the AAML0531 trial) improved outcomes of patients within the low ACS10 score group.^5^ In this report, we further validated the clinical relevance of ACS10 score in a large and independent cohort of pediatric and AYA patients who participated in the COG AAML1031 trial. In that trial, patients with a low ACS10 score, when treated with standard induction (arm A, ADE) had significantly reduced EFS, thus highlighting the association of ACS10 score with outcomes in childhood AML. The ACS10 score was not associated with outcomes in the augmented arm B (ADE + bortezomib), implying therapy augmentation may abrogate the detrimental impact of a low ACS10 score. These results suggest that augmentation of the standard chemotherapy regimen can mitigate the poor outcomes in patients within the ACS10 low score group.

Expanding this work to AYA patients aged 17 to 39 years who participated in Alliance trials, the ACS10 score remained significantly associated with OS in patients with AML. These results are especially promising given that the ACS10 score, until recently, has only been tested in pediatric populations.

As indicated previously, several reports have shown that Black patients with AML have worse outcomes than White patients. We consistently observed that a low ACS10 score, which was associated with poor survival outcomes in response to standard chemotherapy, was abundant in Black trial populations (AML02 and AAML0531 inour previous work^5^; AAML1031 and Alliance in this study). Thus, pharmacogenomic differences between Black and White patients may contribute to the observed disparities with respect to clinical outcomes. Increased abundance of low ACS10 scores among Black patients is attributed to 3 ancestry-associated SNVs that account for these genetic differences and are reflected in the varying allele frequencies observed in populations of African ancestry vs European ancestry.^5^

In summary, the results of this study validated the clinical relevance of the ACS10 score in patients treated with standard induction regimen (consisting of 100mg/m^2^ ara-C) in the AAML1031 clinical trial; observed that individuals with low ACS10 scores seem to benefit from therapy augmentation, such as with intensified ara-C or the addition of gemtuzumab ozogamicin as shown in a previous report^5^ and the use of bortezomib suggested by our current study; and revealed that the proportion of patients with low ACS10 scores, which was associated with poor outcomes, was significantly higher in Black patients. This final finding may provide a potential mechanism for historically poor outcome observed in Black patients compared with White patients with AML. It is also consistent with the previously observed lower CR rates of Black AYA patients when treated with standard induction chemotherapy.^8^ Collectively, this study and prior results suggest that Black patients with low ACS10 scores (which includes approximately 70% of Black patients with AML) may require intensified induction and/or consolidation therapy to overcome differences in ara-C sensitivities. Finally, this study’s expansion and application of pharmacogenomics to adult cohorts highlight its relevance in AML treatment.

The findings of this study suggest the possibility of identifying patients at increased risk of poor response to cytarabine-based induction regimens and offer the opportunity to customize treatment regimens for patients with AML via preemptive ACS10 score assessment. Furthermore, ACS10 SNV genotyping in a clinical setting is highly feasible, as these are germline variants and results can be obtained with quick turnaround time depending on the capabilities of personnel and laboratory equipment. The formula for calculating ACS10 was established and validated in our earlier publications. Based on those findings, along with the results presented in the current study, it appears that the ACS10 score can be used to select the most effective induction regimen for a patient.

Our prior work has shown that augmentation of the standard treatment regimen with agents such as high dose ara-C, gemtuzumab,^5^ or clofarabine provides significant benefit for patients with a low ACS10 score.^32,33^ The present study provides support for bortezomib as an additional augmentation option for this subgroup. These personalized treatment approaches hold promise not only for improving overall outcomes but also for helping to reduce racial disparities in survival outcomes.

However, several challenges remain in translating these findings into prospective clinical practice. Key among them is the integration of genetic data into electronic health records, along with the development of clinical decision support tools, such as alert systems or tailored consult notes to assist clinicians in selecting appropriate personalized treatment regimens. Beyond AML, the findings from this study may have broader implications for other nucleoside analogs that share activation pathways with ara-C and are used in the treatment of hematological malignant neoplasms. Pharmacogenomics-guided personalized remission induction regimens open strategies to explore similar approaches for other antileukemic agents across other hematological malignant neoplasms.

### Limitations

This study has several limitations. First, genetically determined ancestry for the AAML1031 cohort was not available. Although AAML1031 represents one of the largest pediatric AML trials, the number of Black patients, particularly within the high ACS10 score group, was limited, thus restricting the ability to perform meaningful statistical analyses in this subgroup. The observed racial differences in ACS10 score distribution underscore the need for future prospective clinical trials to enroll patients with different racial and ethnic backgrounds. Second, the inclusion of data from 9 individual Alliance trials, conducted over a 20-year period, introduces potential variability related to evolving treatment protocols, advances in supportive care, and other trial-specific factors. Despite the inherent heterogeneity across the included trials, we did observe an association between the ACS10 score and clinical outcomes, implying the potential clinical relevance in the context of standard induction regimens. The integration of the ACS10 score into a future prospective clinical trial is warranted and represents a critical step toward advancing pharmacogenomics-guided treatment strategies in AML. Validation of its clinical relevance in a clinical trial setting would support its use in guiding treatment decisions, enabling clinicians to optimize therapeutic approaches based on an individual’s genetic profile.

## Conclusions

In this cohort study, a low ACS10 score among pediatric patients with AML was associated with worse EFS; among AYA patients with AML, a low ASC10 score was associated with worse OS. Additionally, Black patients were more likely than White patients to have a low ACS10 score. These findings highlight the relevance of the ACS10 score in personalizing treatment of AML across ages and racial and ethnic groups.

## Notes

Supplement 1. **eFigure 1.** EFS and OS by Treatment Arms in 717 Patients from AAML1031 Trial Included in This Study **eFigure 2.** Frequency Distribution of ACS10 Scores in Patients From AAML1031 Trial **eFigure 3.** EFS and OS by ACS10 Score Groups in All Patients and Non-HSCT Patients in Arm A and B of AAML1031 Trial **eFigure 4.** EFS and OS by ACS10 Score in All Patients in Arm B and Patients in Arm B Who Did Not Receive HSCT **eFigure 5.** Kaplan Meier Survival Curves for Event-Free Survival (EFS) Using Transplant as a Time-Dependent Covariate in Both Treatment Arms With All Patients and in Standard and High-Risk Group Patients **eFigure 6.** Forest Plot Showing OS by ACS10 Score in Patients in Arm A and Arm B **eFigure 7.** Survival Outcome by Treatment Arm A vs Arm B Among Black Patients **eFigure 8.** Association of ACS10 Score Groups (High vs Low) With Intracellular Ara-CTP Levels in Patients in the AML97 Clinical Trial **eTable 1.** SNVs and Genes That Are Part of the ACS10 Score **eTable 2.** Patient Characteristics Summary for AAML1031 Trial in Whole Cohort and by ACS10 Groups **eTable 3.** Characteristics for De Novo AML Patients Younger Than 40 Years in Alliance Trials in Whole Cohort and by ACS10 Groups

Supplement 2.
Data Sharing Statement
