# Supplementary material for: Cytarabine Pharmacogenomics and Outcomes Among Children and Young Adults With Acute Myeloid Leukemia
Source: JAMA Netw Open. 2025 Jun 23;8(6):e2516296. doi: 10.1001/jamanetworkopen.2025.16296 (PMC12186577; doi:10.1001/jamanetworkopen.2025.16296)
Supplement: Supplement 1. — eFigure 1. EFS and OS by Treatment Arms in 717 Patients from AAML1031 Trial Included in This Study eFigure 2. Frequency Distribution of ACS10 Scores in Patients From AAML1031 Trial eFigure 3. EFS and OS by ACS10 Score Groups in All Patients and Non-HSCT Patients in Arm A and B of AAML1031 Trial eFigure 4. EFS and OS by ACS10 Score in All Patients in Arm B and Patients in Arm B Who Did Not Receive HSCT eFigure 5. Kaplan Meier Survival Curves for Event-Free Survival (EFS) Using Transplant as a Time-Dependent Covariate in Both Treatment Arms With All Patients and in Standard and High-Risk Group Patients eFigure 6. Forest Plot Showing OS by ACS10 Score in Patients in Arm A and Arm B eFigure 7. Survival Outcome by Treatment Arm A vs Arm B Among Black Patients eFigure 8. Association of ACS10 Score Groups (High vs Low) With Intracellular Ara-CTP Levels in Patients in the AML97 Clinical Trial eTable 1. SNVs and Genes That Are Part of the ACS10 Score eTable 2. Patient Characteristics Summary for AAML1031 Trial in Whole Cohort and by ACS10 Groups eTable 3. Characteristics for De Novo AML Patients Younger Than 40 Years in Alliance Trials in Whole Cohort and by ACS10 Groups [file jamanetwopen-e2516296-s001.pdf]

Supplemental Online Content

Marrero RJ, Shastri VM, Nicolet D, et al. Cytarabine Pharmacogenomics and Outcomes Among Children and Young Adults With Acute Myeloid Leukemia. *JAMA Netw Open.* 2025;8(6):e2516296. doi:10.1001/jamanetworkopen.2025.16296

Supplement 1. eFigure 1. EFS and OS by Treatment Arms in 717 Patients from AAML1031 Trial Included in This Study

eFigure 2. Frequency Distribution of ACS10 Scores in Patients From AAML1031 Trial

eFigure 3. EFS and OS by ACS10 Score Groups in All Patients and Non-HSCT Patients in Arm A and B of AAML1031 Trial

eFigure 4. EFS and OS by ACS10 Score in All Patients in Arm B and Patients in Arm B Who Did Not Receive HSCT

eFigure 5. Kaplan Meier Survival Curves for Event-Free Survival (EFS) Using Transplant as a Time-Dependent Covariate in Both Treatment Arms With All Patients and in Standard and High-Risk Group Patients

eFigure 6. Forest Plot Showing OS by ACS10 Score in Patients in Arm A and Arm B

eFigure 7. Survival Outcome by Treatment Arm A vs Arm B Among Black Patients

eFigure 8. Association of ACS10 Score Groups (High vs Low) With Intracellular Ara-CTP Levels in Patients in the AML97 Clinical Trial

eTable 1. SNVs and Genes That Are Part of the ACS10 Score

eTable 2. Patient Characteristics Summary for AAML1031 Trial in Whole Cohort and by ACS10 Groups

eTable 3. Characteristics for De Novo AML Patients Younger Than 40 Years in Alliance Trials in Whole Cohort and by ACS10 Groups

This supplemental material has been provided by the authors to give readers additional information about their work.

© 2025 Marrero RJ et al. *JAMA Network Open*.

eFigure 1. EFS and OS by Treatment Arms in 717 Patients from AAML1031 Trial Included in This Study

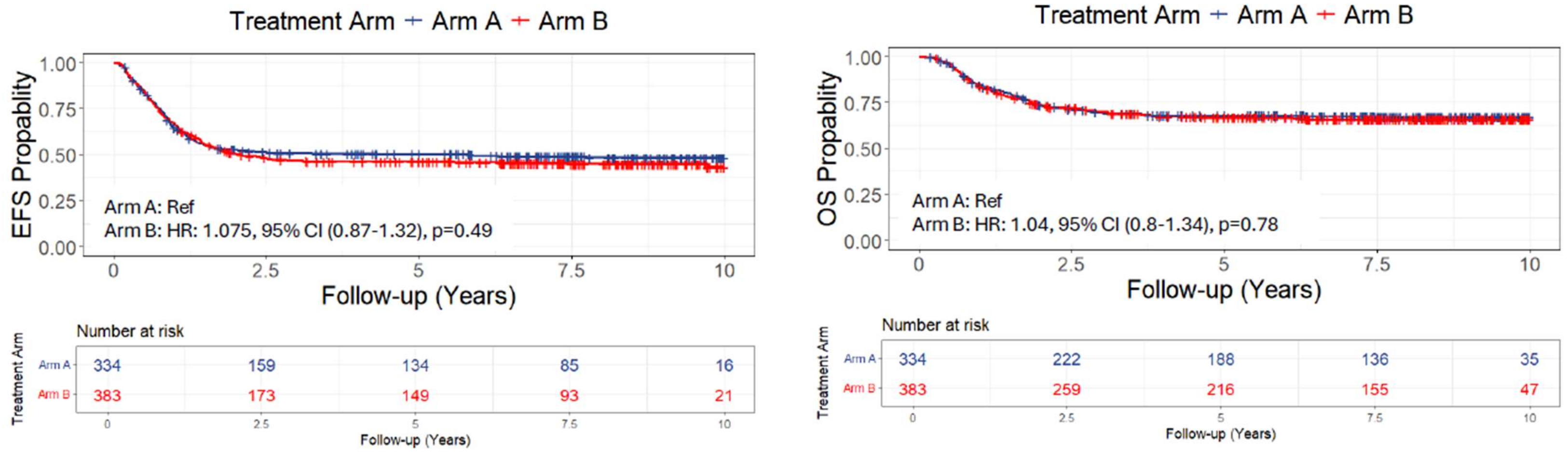

Arm A: blue; Arm B : red.

© 2025 Marrero RJ et al. *JAMA Network Open*.

eFigure 2. Frequency Distribution of ACS10 Scores in Patients From AAML1031 Trial

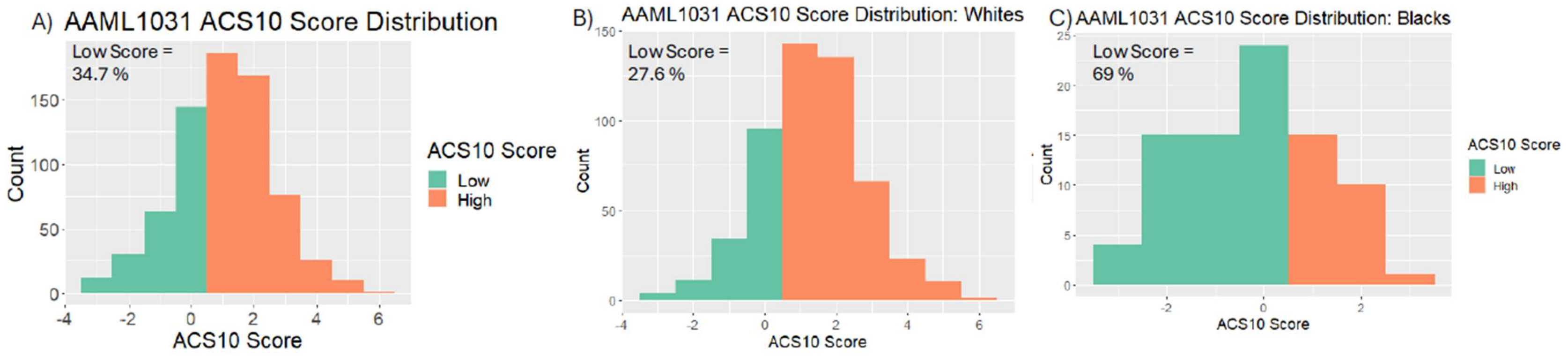

A) all patients, B) White patients and C) Black patients

© 2025 Marrero RJ et al. *JAMA Network Open*.

eFigure 3. EFS (A and C) and OS (B and D) by ACS10 Score Groups in All Patients (N=717) and Non-HSCT Patients (N=577) in Arm A and B of AAML1031 Trial

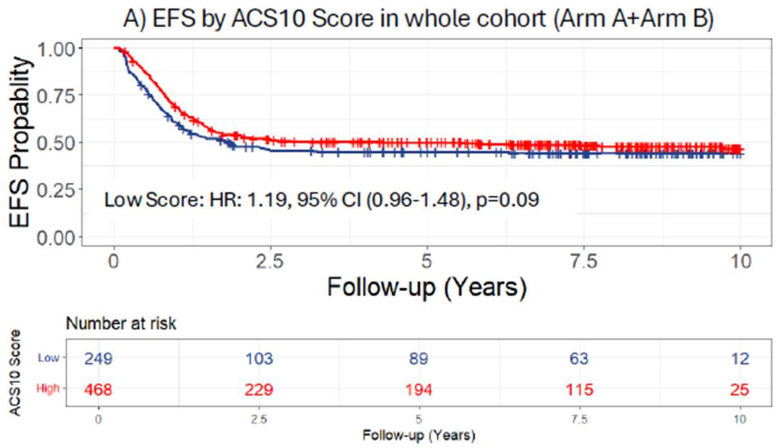

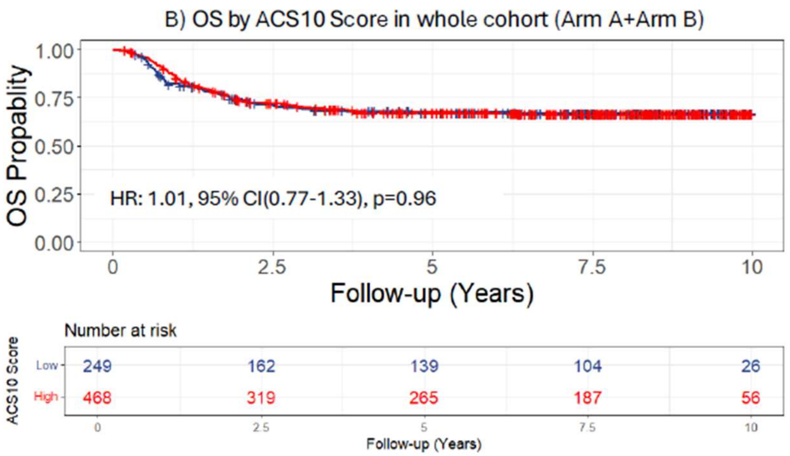

© 2025 Marrero RJ et al. *JAMA Network Open*.

eFigure 3 (cont.)

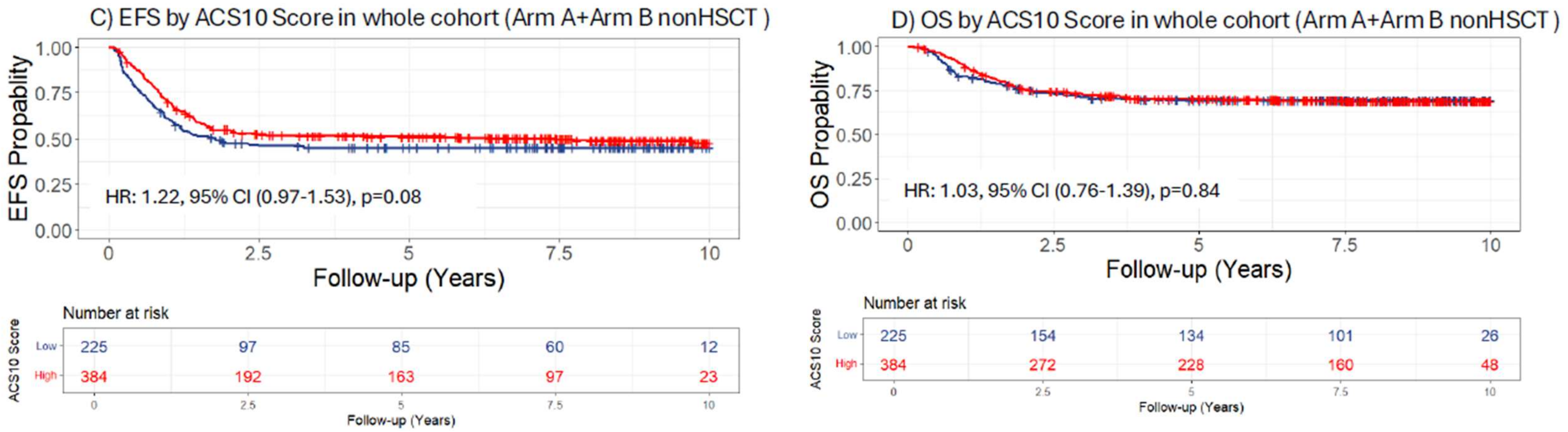

ACSL10^{Low}; Blue; ACS10^{High} : red; ACS10 High Score is used as reference group

© 2025 Marrero RJ et al. *JAMA Network Open*.

eFigure 4. EFS and OS by ACS10 Score in All Patients in Arm B and Patients in Arm B Who Did Not Receive HSCT

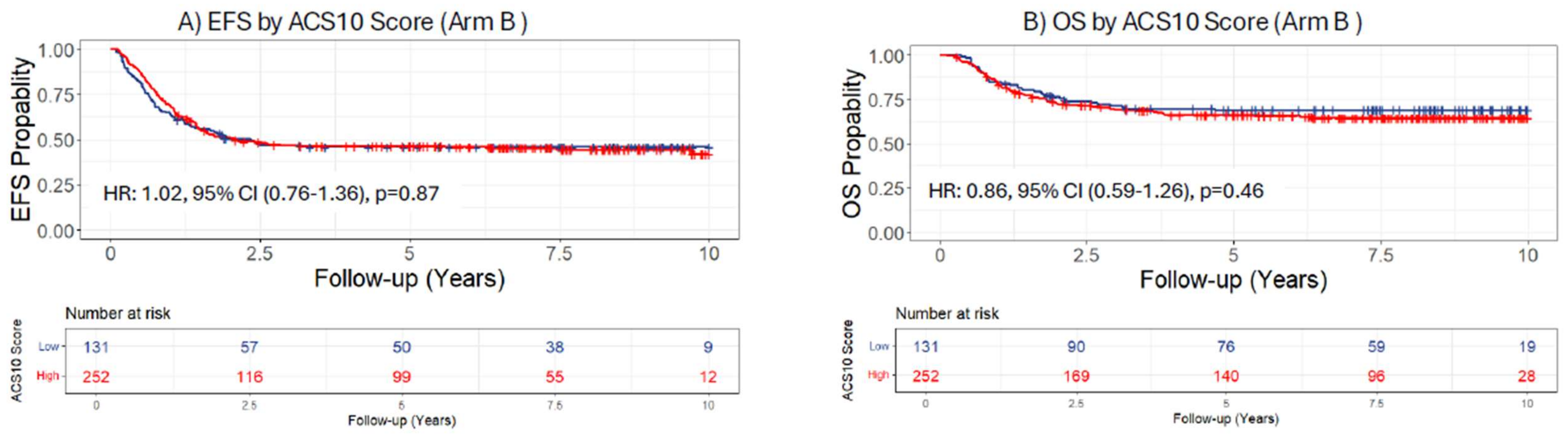

© 2025 Marrero RJ et al. *JAMA Network Open*.

eFigure 4 (cont.)

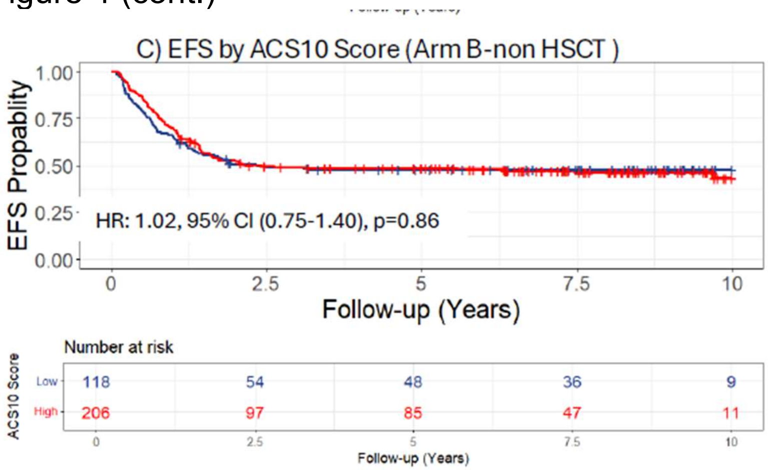

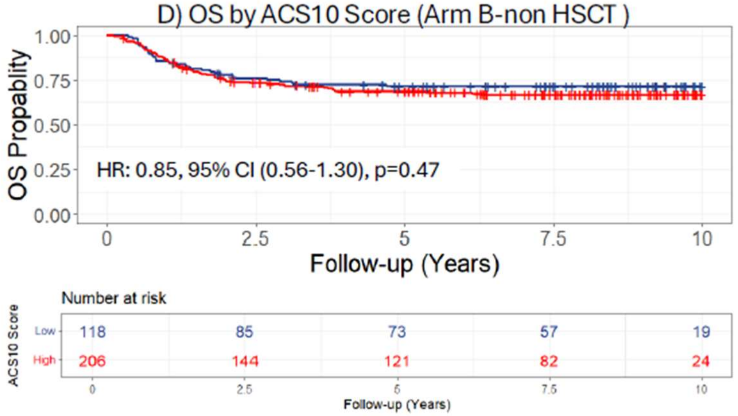

t.)

ACSL10^{Low}; Blue; ACS10^{High} : red; ACS10 High Score is used as reference group.

© 2025 Marrero RJ et al. *JAMA Network Open*.

eFigure 5. Kaplan Meier Survival Curves for Event-Free Survival (EFS) Using Transplant as a Time-Dependent Covariate in Both Treatment Arms With All Patients and in Standard and High-Risk Group Patients

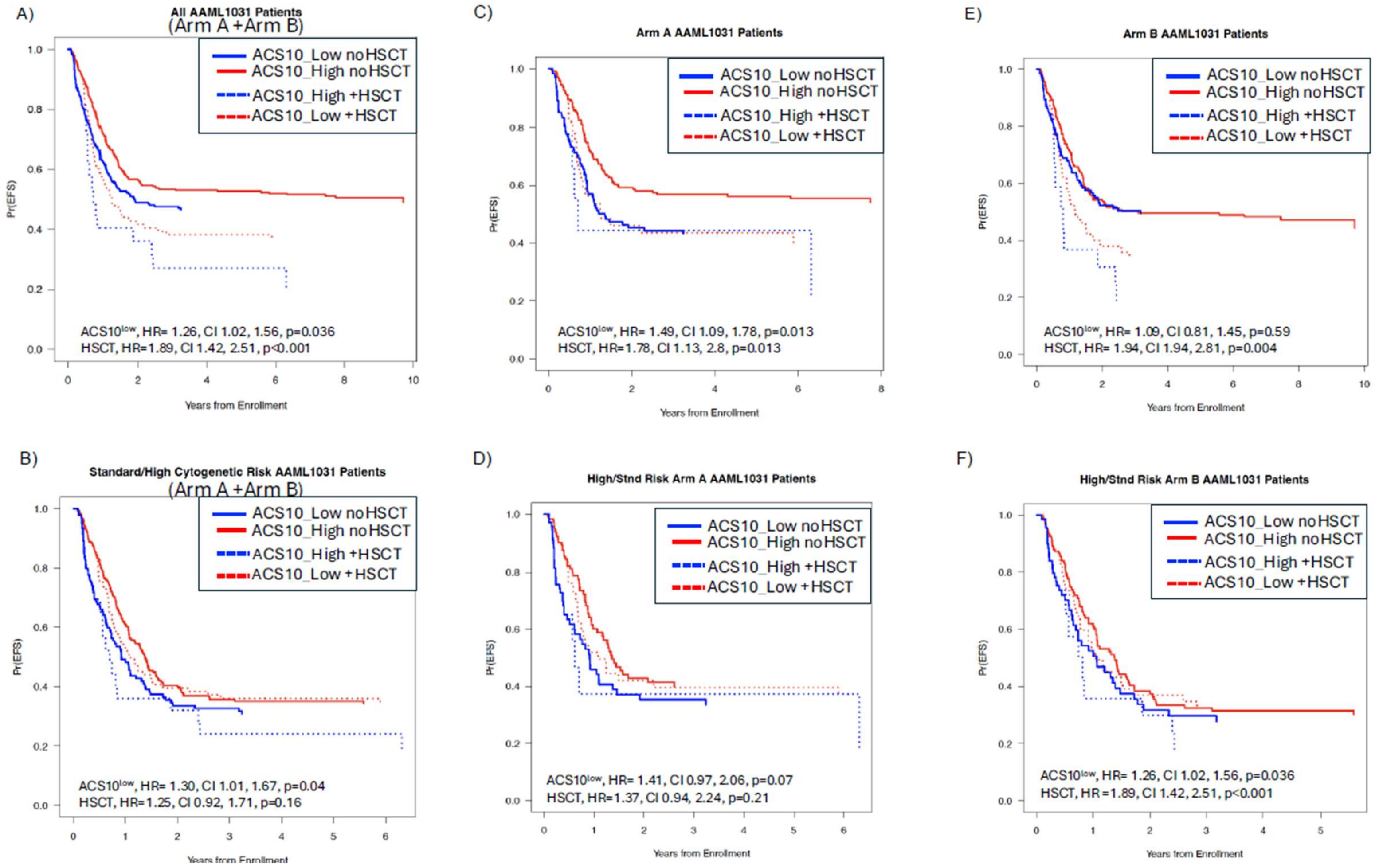

© 2025 Marrero RJ et al. *JAMA Network Open*.

eFigure 6. Forest Plot Showing OS by ACS10 Score in Patients in Arm A and Arm B

A) Multivariable Analysis – Arm A: OS by ACS10 Score

Variable		N	Hazard ratio		p
**ACS10_Group**	High	213		Reference	
	Low	117		1.12 (0.73, 1.74)	0.600
**Risk_Group**	Low	143		Reference	
	Standard	174		4.07 (2.47, 6.69)	<0.001
	High	13		3.63 (1.44, 9.14)	0.006
**Race**	White	249		Reference	
	Black	39		1.89 (1.06, 3.39)	0.031
	Asian	14		1.57 (0.63, 3.90)	0.334
	Unknown	28		1.14 (0.54, 2.37)	0.733
**WBC**	<30 (G/L)	210		Reference	
	>= 30 (G/L)	120		1.76 (1.17, 2.65)	0.007
**Age**	<10 (Years)	169		Reference	
	>= 10 (Years)	161		1.43 (0.95, 2.15)	0.086
			1 2 5		

B) Multivariable Analysis – Arm B: OS by ACS10 Score

Variable		N	Hazard ratio		p
**ACS10_Group**	High	247		Reference	
	Low	127		0.83 (0.56, 1.25)	0.4
**Risk_Group**	Low	154		Reference	
	Standard	192		5.87 (3.46, 9.97)	<0.001
	High	28		5.01 (2.38, 10.56)	<0.001
**Race**	White	264		Reference	
	Black	42		1.38 (0.80, 2.38)	0.2
	Asian	19		1.34 (0.61, 2.95)	0.5
	Unknown	49		0.76 (0.43, 1.37)	0.4
**WBC**	<30 (G/L)	216		Reference	
	>= 30 (G/L)	158		0.90 (0.62, 1.31)	0.6
**Age**	<10 (Years)	192		Reference	
	>= 10 (Years)	182		1.11 (0.76, 1.62)	0.6
			0.5 1 2 5 10		

© 2025 Marrero RJ et al. *JAMA Network Open*.

eFigure 7. Survival Outcome by Treatment Arm A vs Arm B Among Black Patients

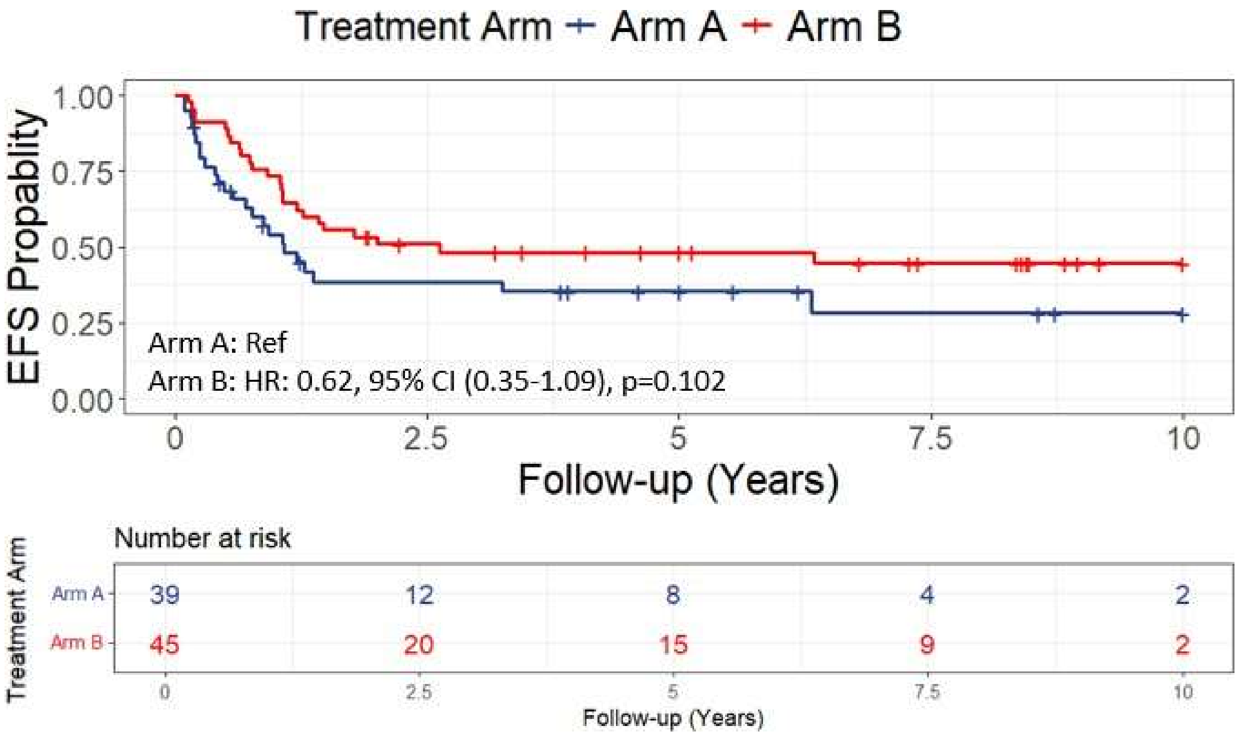

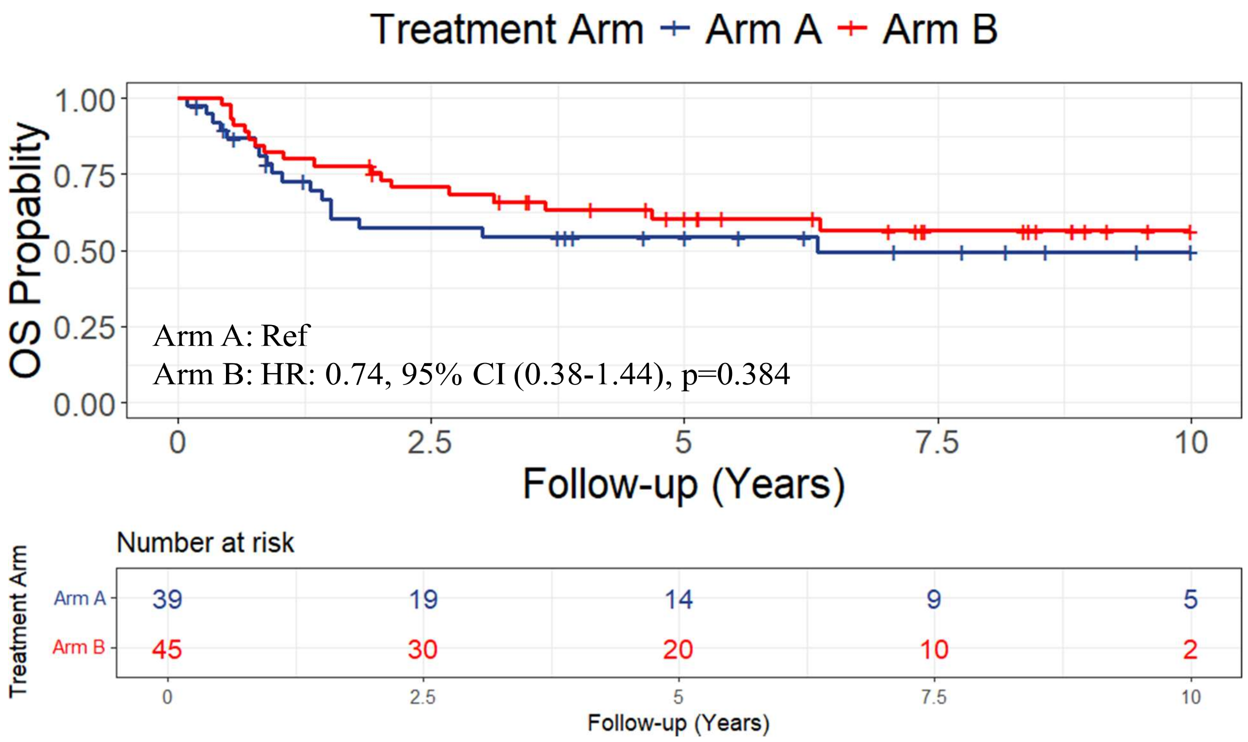

Arm A: Blue; Arm B : red; Arm A is used as reference group

© 2025 Marrero RJ et al. *JAMA Network Open*.

eFigure 8. Association of ACS10 Score Groups (High vs Low) With Intracellular Ara-CTP Levels in Patients in the AML97 Clinical Trial

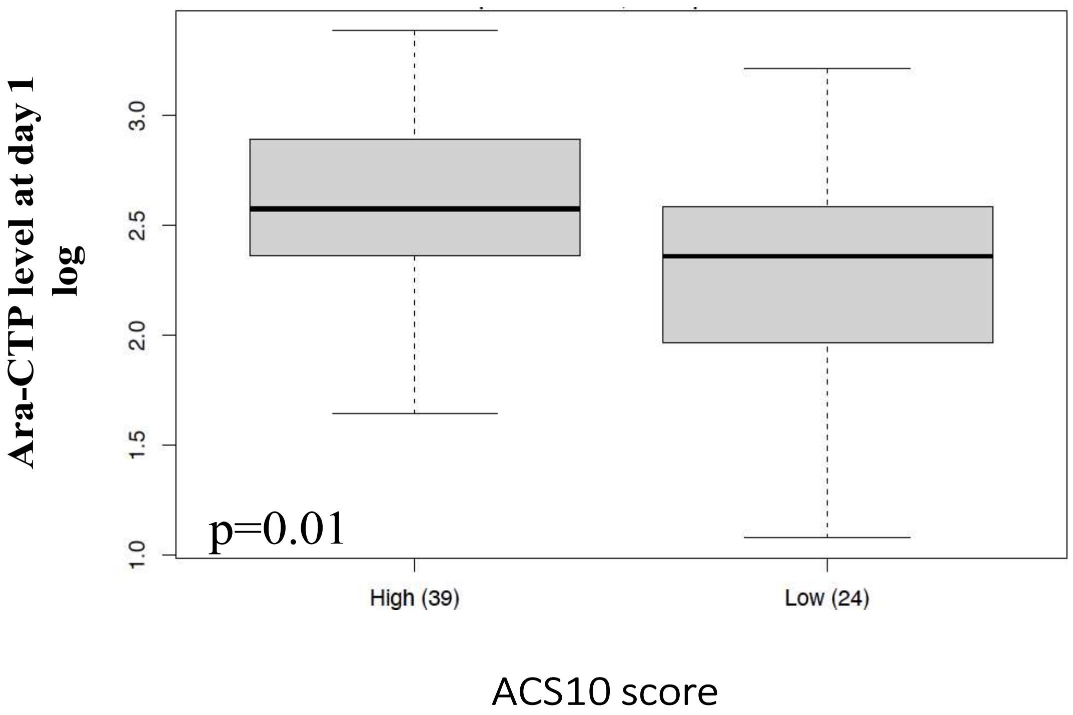

Ara-CTP levels were measured in bone marrow aspirates obtained 24hr post initiation of ara-C infusion as described previously (Elsayed et al, 2018). ACS10 was calculated previously developed score (Elsayed et al, 2022). P value represents Wilcoxon test.

© 2025 Marrero RJ et al. *JAMA Network Open*.

eTable 1. SNVs and Genes That Are Part of the ACS10 Score

Gene	SNP	Role in Ara-C metabolic Pathway	Initial association	Reference	Individual SNP Genotype scores [ACS10 =Sum of genotype scores]
CDA	**rs10916819**	**Inactivation**	MRD1	PMID:34990262	AA = 0, AG/GG = -1
CMPK1	**rs17103168**	**Activation**			AA = 0, AG/GG = 1
NME4	**rs5841**	**Activation**			CC = 0, CT/TT = 1
SLC29A1	**rs2396243**	**Uptake transporter**	EFS/OS		GG = 0, AG = -1, AA = -2
CMPK1	**rs1044457**	**Activation**			CC = 0, CT/TT = 1
RRM2	**rs1138729**	**Impacts Ara-C activation by regulating cellular dCTP levels**			AA = 0, AG/GG = -1
DCK	**rs4643786**	**Activation**	intracellular ara-CTP levels	PMID :30088438	TT = 0, CT = -1, CC = -2
RRM1	**rs11030918**	**regulating cellular dCTP levels**			TT/CT = 0, CC = 1
CTPS1	**rs12067645**				GG = 0, AG = 1, AA = 2
SLC28A3	**rs17343066**	**Uptake transporter**			GG/AG = 0, AA = 1

SNPs associated with deterimental outcome are in grey.

© 2025 Marrero RJ et al. *JAMA Network Open*.

eTable 2. Patient Characteristics Summary for AAML1031 Trial in Whole Cohort and by ACS10 Groups

	AAML1031 (N=717) Arm A and Arm B Only	ACS10 Low Score Group (N=249)	ACS10 High Score Group (N=468)	P (low vs. high ACS10)
**Age (continuous)**	9.8 (0.04-29.2)	9.3 (0.17-29.2)	10.0 (0.04-28.3)	0.171
**Age Group, years**				
< 10	370 (51.6%)	140 (56.2%)	230 (49.1%)	0.117
≥ 10	347 (48.4%)	109 (43.8%)	238 (50.9%)	
**Sex**				
Male	379 (52.9%)	140 (56.2%)	239 (51.1%)	0.188
Female	338 (47.1%)	109 (43.8%)	229 (48.9%)	
**Risk group**				
Low	297 (41.4%)	105 (42.2%)	192 (41.0%)	0.958
Standard	366 (51.0%)	124 (49.8%)	242 (51.7%)	
High	41 (5.7%)	15 (6.0%)	26 (5.5%)	
Missing	13 (1.8%)	5 (2.0%)	8 (1.7%)	
**Race**				
Black	84 (11.7%)	58 (23.2%)	26 (5.5%)	**<0.001**
Other	78 (10.9%)	32 (12.9%)	46 (9.8%)	
White	522 (72.8%)	144 (57.8%)	378 (80.8%)	
Asian	33 (4.6%)	15 (6.0%)	18 (3.8%)	
**Ethnicity**				
Hispanic or Latino	135 (18.8%)	53 (21.3%)	82 (17.5%)	0.468
Not Hispanic or Latino	559 (78.0%)	188 (75.5%)	371 (79.3%)	
Unknown	23 (3.2%)	8 (3.2%)	15 (3.2%)	
**Cytogenetics Group**				
11q23 rearrangements	168 (23.4%)	62 (24.9%)	106 (22.6%)	0.494
inv(16)	75 (10.4%)	28 (11.2%)	47 (10.0%)	
Normal	148 (20.6%)	48 (19.3%)	100 (21.4%)	
Other	204 (28.5%)	63 (25.3%)	141 (30.1%)	
t(8:21)	122 (17.0%)	48 (19.3%)	74 (15.8%)	
**WBC group (G/L)**				
< 50	516 (72.0%)	178 (71.5%)	338 (72.0%)	0.834
≥ 50	201 (28.0%)	71 (28.5%)	130 (28.0%)	
**WBC (continous)**	53.5 (0.6-918.5)	54.5 (1.0-550.0)	52.9 (0.6-918.5)	0.819
**Treatment arm**				
Arm A	334 (46.6%)	118 (47.4%)	216 (46.2%)	0.752
Arm B	383 (53.4%)	131 (52.6%)	252 (53.8%)	
**HSCT Group**				
SCT in CR	95 (13.2%)	20 (8.0%)	75 (16.0%)	**0.008**
No SCT in CR	577 (80.5%)	215 (86.3%)	362 (77.4%)	
Unknown	45 (6.3%)	14 (5.6)	31 (6.6%)	

Notes: 1) Continuous variables are presented as mean with range and frequency of categorical variables is presented as a percentage ; 2) Risk group based on cyto-molecular features; 3) P values < 0.05 indicated in bold

© 2025 Marrero RJ et al. *JAMA Network Open*.

eTable 3. Characteristics for De Novo AML Patients Younger Than 40 Years in Alliance Trials in Whole Cohort and by ACS10 Groups

	**Whole cohort (N=369)**	**ACS10 Low Score Group (N=112)**	**ACS10 High Score Group (N=257)**	**P (low vs. high ACS10)**
**Age**				0.65
	30 (17-39)	30 (18-39)	30 (17-39)	
**Gender**				0.36
Male	196 (53%)	55 (49%)	141 (55%)	
Female	173 (47%)	57 (51%)	116 (45%)	
**Race**				**<0.001**
White	288 (78%)	64 (60%)	224 (89%)	
Black	32 (9%)	27 (24%)	5 (2%)	
Hisp	21 (6%)	8 (7%)	13 (5%)	
Asian	7 (2%)	3 (3%)	4 (2%)	
Other	11 (3%)	4 (4%)	7 (3%)	
**HSCT status**				1.00
Yes HSCT	118 (32%)	36 (32%)	82 (32%)	
No HSCT	251 (68%)	76 (68%)	175 (68%)	
**ELN 2022 group**				0.75
Favorable	132 (42%)	40 (45%)	92 (41%)	
Intermediate	97 (31%)	25 (28%)	72 (32%)	
Adverse	85 (27%)	24 (27%)	61 (27%	
**Protocol**				
CALGB 9022	9 (2%)	2 (2%)	7 (3%)	
CALGB 9120	3 (1%)	2 (2%)	1(<1%)	
CALGB 9222	41 (11%)	9 (8%)	32 (12%)	
CALGB 9621	68 (18%)	24 (21%)	44 (175)	
CALGB 10503	92 (25%)	27 (24%)	65 (25%)	
CALGB 10603	29 (8%)	12 (11%)	17 (7%)	

© 2025 Marrero RJ et al. *JAMA Network Open*.

CALGB 10801	11 (3%)	5 (4%)	6 (2%)	
CALGB 19808	116 (31%)	31 (28%)	85 (33%)	

© 2025 Marrero RJ et al. *JAMA Network Open*.
