# Supplementary material for: Cytarabine Pharmacogenomics and Outcomes Among Children and Young Adults With Acute Myeloid Leukemia
Source: JAMA Netw Open. 2025 Jun 23;8(6):e2516296. doi: 10.1001/jamanetworkopen.2025.16296 (PMC12186577; doi:10.1001/jamanetworkopen.2025.16296)
Supplement: Supplement 2. — Data Sharing Statement [file jamanetwopen-e2516296-s002.pdf]

Data Sharing Statement

Marrero. Cytarabine Pharmacogenomics and Outcomes Among Children and Young Adults With Acute Myeloid Leukemia. *JAMA Netw Open*. Published June 16, 2025. doi:10.1001/jamanetworkopen.2025.16296

Data
Data available: Yes
Data types: Deidentified participant data
How to access data: Drs. Lamba and Eisfeld had full access to all the data in the study and take responsibility for the integrity of the data and the accuracy of the data analysis. Deidentified participant data will be made available at the time of publication for a period of five years after the publication date. Anyone may request the data for any purpose and will be subject to a signed data access agreement. Proposals for access should be sent to Jatinder K Lamba jatinderklamba@ufl.edu
When available: beginning date: 03-01-2026

Supporting Documents
Document types: None

Additional Information
Who can access the data: researchers whose proposed use of the data has been approved
Types of analyses: genotype based associated
Mechanisms of data availability: after approval of a proposal, or with a signed data access agreement
